# The evaluation of alternative method of ferrous ions assessment in pharmaceutical preparations

**DOI:** 10.1007/s00706-018-2147-5

**Published:** 2018-02-13

**Authors:** Anna Lisik, Anna Prescha, Levent E. Cavlaz, Halina Grajeta, Witold Musiał

**Affiliations:** 1 Pharmaceutical Faculty, Department of Physical Chemistry, Wroclaw Medical University, Borowska 211, 50-556, Wroclaw, Poland; 2 Pharmaceutical Faculty, Department of Bromatology and Dietetics, Wroclaw Medical University, Borowska 211, 50-556, Wroclaw, Poland; 3 Pharmaceutical Faculty, Department of Pharmacology, Ankara University, Ankara, Turkey

**Keywords:** Drug research, Cations, Ferrous ions, Electrochemistry, Atomic absorption spectrometry, Validation

## Abstract

**Abstract:**

The atomic absorption spectrometry (AAS) method is one of the most accessible procedures for ferrous ions testing in various compositions including pharmaceutical preparations. The aim of the study was to develop and partially validate analytical method which could be an excellent alternative to the routine procedure performed within dissolution studies. Electric conductivity is simple, fast, and hassle-free method. The samples during dissolution process were measured using conductivity probe in entire dissolution assessment protocol. The conductivity results were compared to data obtained from AAS. The dissolution studies were performed according to modified pharmacopoeial standards, in 900 cm^3^ of purified water as an acceptor medium, at 37 °C, until the achievement of an equilibrium state for every tested composition. Validity study of the developed method confirmed acceptable linearity of obtained calibration plots (*r*^2^ > 0.9553). Linearity at 100% level was found to be 100.59, 97.49, and 94.82, respectively, for drug compositions A, B, and C. Precision results were 100.45, 95.97, and 95.73, respectively, for A, B, and C, with RSD below 2% between all samples in all above mentioned formulations. The drug composition D hindered the proper validation of the method due to the high variability between samples. The method has acceptable performance features for evaluation of three of four solid drug composition containing ferrous ions.

**Graphical abstract:**

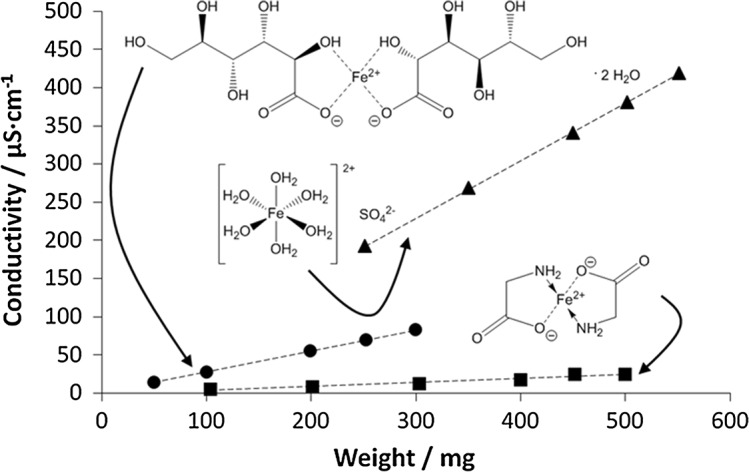

## Introduction

Ferrous ions perform several important features in the human internal environment, i.a., are crucial for the active structure of hemoglobin, effect as resistance factors of the immune system [1], and influence the growth human body tissues. Deficiency of this microelement is one of the most common disorders in global scale; ca 3.5 billion of humans, most of them from developed countries, is suffering the iron deficiency, including anemia [2]. Iron has been registered in many different forms, and is used to treat the iron deficiency and related disorders [3]. Adequate daily supplementation may be sufficient to prevent severe iron deficiency, particularly in pregnant women and children [4, 5]. The variety of iron compounds is administered, enclosing ferric and ferrous forms of elemental iron as salts, complexes, hydrates, chelates, and iron ions linked to polymeric carriers [6, 7]. In present study, three different forms of ferrous ions: ferrous sulfate heptahydrate, ferrous gluconate dehydrate, and iron bis-glycinate chelate were compared. Ferrous sulfate heptahydrate and ferrous gluconate are widely and effectively applied in iron deficiency treatment [8]. The iron bis-glycinate chelate, known as ferrochel, has rather narrow group of recipients. However, there is abundant number of publications suggesting beneficial supplementation using the chelated form, comparing to the ferrous salts [9].

Atomic absorption spectrometry (AAS) is a quantitative, highly sensitive analytical method that enables the determination of a variety of macro- and microelements, notably metal cations, in aqueous, solid, and gaseous samples. The phenomenon of radiation absorption in a specific wavelength by the free metal atoms [10] enables development of numerous methods for quantification of ferrous ions via atomic absorption spectrometry. The procedures may be performed in various types of samples, e.g., in environmental objects: water, soil, or sewage, but numerous methods are dedicated to medicinal products, as well as to foods containing dietary supplements [11–14]. The ability of a solution to conduct electric current gives the occasion for the measurement of the concentration of ionized compounds in polar solutions, particularly in aqueous solutions [15]. The common applications of electric conductivity to ions quantification include fast determination of metal ions concentrations in waste water [16], some studies interpreted the biogeochemical reactivity of water, using conductivity measurements [17]. The present research evaluates the electric conductivity as the new method for fast assessment of dissolution of preparations containing iron in ionized form. Validation is an activity, intended to document the repeatability of an analytical method and adherence of the method to the strictly defined acceptance criteria, governed by ICH guidelines [18]. The ICH validation guidelines—for the method determining the drug content—include specificity, linearity, accuracy, sensitivity, precision including intermediate precision, and robustness. The validation process is one of the most important procedures in the pharmaceutical industry, leading to the quality assurance of medicinal products with high risk potential. The manufactured pharmaceuticals are inspected due to global guidelines, before the release to the market circulation.

The aim of this work was to develop, evaluate, and validate the new method of release of ferrous ions from solid dosage forms, using electric conductivity and compare it to routine atomic absorption spectrometry method developed in our University, applying the selected guidelines of ICH.

## Results and discussion

**Fig. 1:**
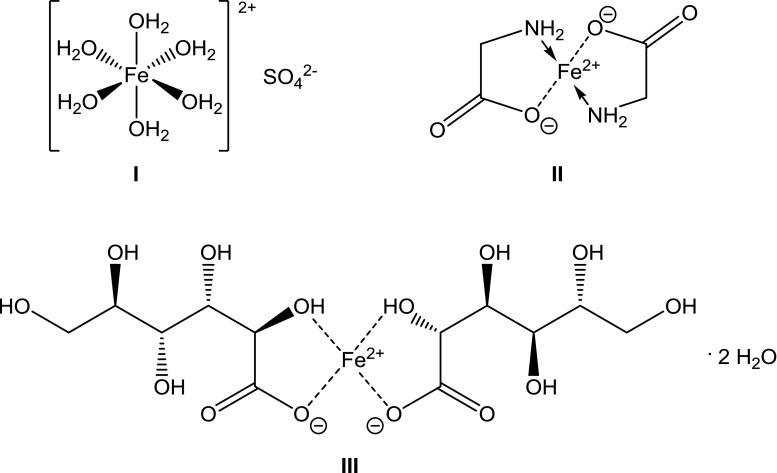
Iron structures in the evaluated compositions: I—ferrous sulfate heptahydrate in compositions A and B, II—iron bis-glycinate chelate in composition D, III—iron(II) gluconate in composition C

**Fig. 2:**
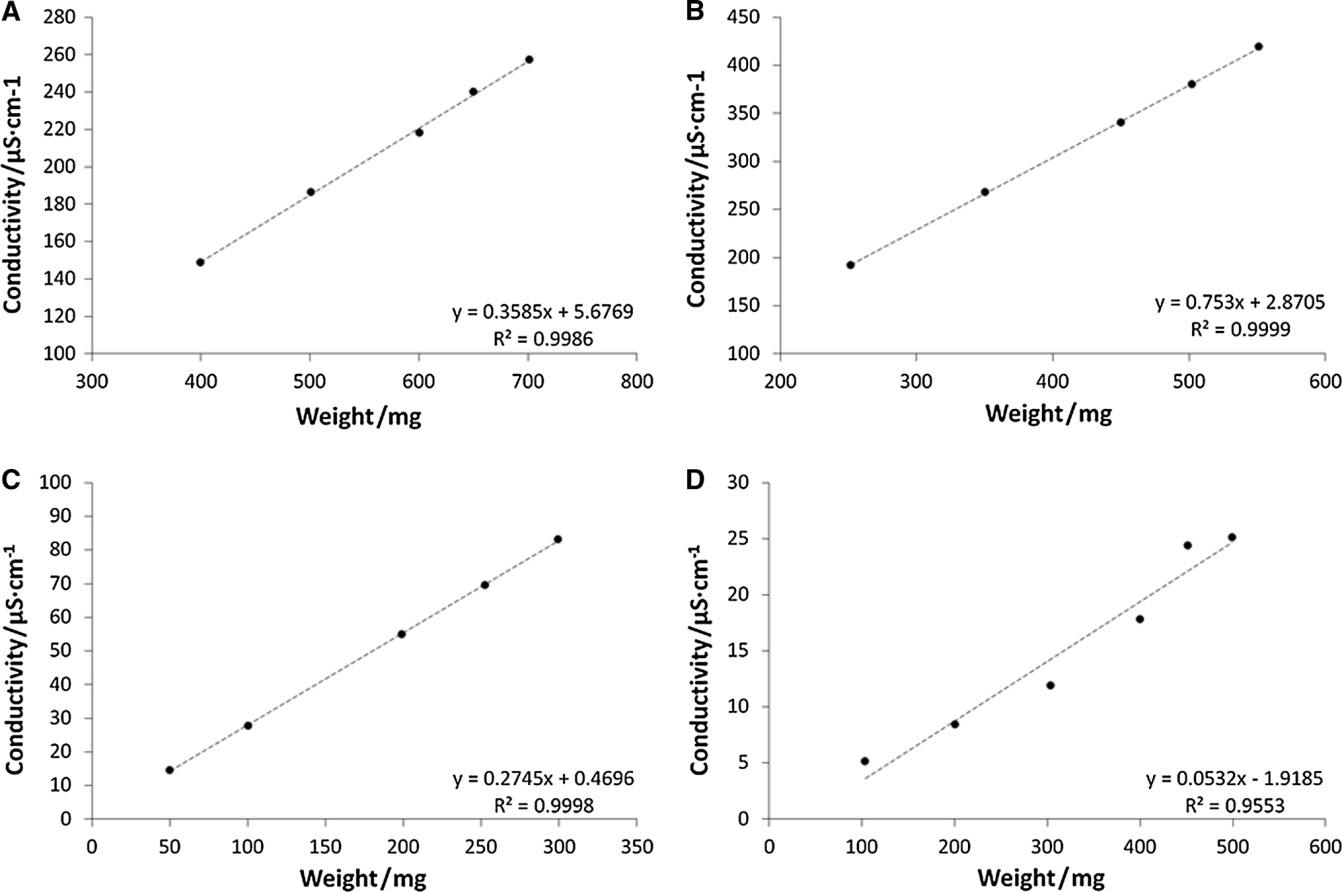
Calibration plots: A—composition A, B—composition B, C—composition C, D—composition D, for measurements performed via electric conductivity

**Fig. 3:**
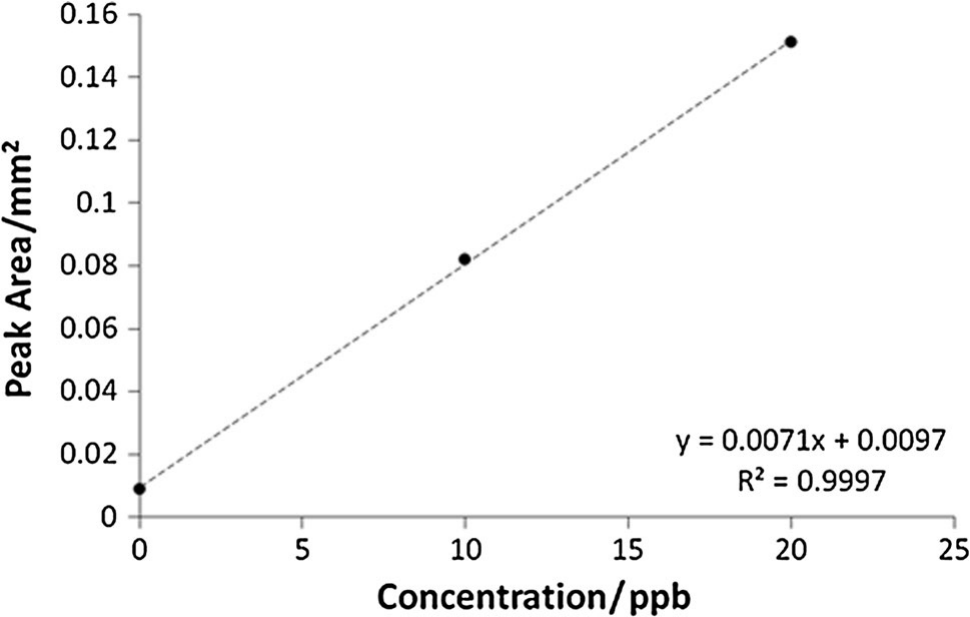
Calibration plot for atomic absorption spectrometry method

AAS method is a routine method for quantification of ferrous ions concentration in the various samples, and presented hereby procedure was developed, evaluated, and validated in our Pharmaceutical Faculty. The method was applied on four various compositions containing ferrous ions (Fig. 1). The results are presented in Table 1. The data reflect the total amount of ferrous ions released respectively from compositions A–D, and assessed when the plateau phase was acquired in the conditions of dissolution test, performed according to the pharmacopoeial standards [19]. The results of respective calibration plots for conductivity and ASA measurements are given in the Figs. 2 and 3.

**Table 1:** Percentage of released API evaluated via AAS measurements at acquired plateau level

Sample no.	Released API/%			
	Composition A	Composition B	Composition C	Composition D
1	76.09	71.65	108.47	17.16
2	59.48	82.06	92.51	26.78
3	71.80	87.77	103.10	61.43
4	67.20	75.68	92.81	53.68
5	66.39	93.76	95.49	78.71
6	61.85	101.17	99.52	42.60
Mean	67.14	85.35	98.65	46.73
RSD	6.15	11.13	6.31	22.70

*API* active pharmaceutical ingredient, *A* included 105.0 mg of ferrous ions as ferrous sulfate heptahydrate and 350 μg of folic acid in the tablet form with prolonged release of bioactives, *B* contained 100.0 mg of iron as ferrous sulfate heptahydrate and 60 mg of ascorbic acid, similarly as in composition A in the form of tablets with prolonged release, *C* included iron as ferrous gluconate dihydrate corresponding to 23.2 mg of iron, *D* included iron as iron bis-glycinate chelate—28.0 mg, folic acid—400 µg, ascorbic acid—40.0 mg, vitamin B6—1.4 mg and vitamin B12—2.5 mg in the form of capsules, *AAS* atomic absorption spectrometry, *RSD* relative standard deviation

The results of AAS procedure in this case were insufficient, presumably due to complex composition of the tablet matrix and capsule filling. The soluble residues of excipients that have been subjected to pyrolysis could have adverse effects on the recorded readings. The data could be influenced by iterative dilution of the samples induced via high primary content of the element in the solution. The main aim of this work was to evaluate and partially validate the new method of ferrous ions release using electric conductivity which could be a practical alternative in the future research. There is a lack of bibliographic data on previous studies aiming measurement of ferrous ions concentration in the process of dissolution of solid forms of drugs, and the literature cannot support the optimization of the analytical method. The initial stage of this research implemented four solid, peroral, commercially available compositions of iron. The preliminary data are presented in Table 2, and arranged as the data in Table 1.

**Table 2:** Percentage of released API evaluated via electric conductivity measurements at acquired plateau level

Sample no.	Released API/%			
	Composition A	Composition B	Composition C	Composition D
1	98.16	97.87	94.25	86.60
2	106.11	100.31	92.49	76.34
3	103.54	95.81	97.24	74.15
4	100.07	97.78	93.22	93.68
5	98.02	99.26	96.37	55.96
6	97.64	93.93	94.26	54.38
Mean	100.59	97.49	94.64	73.52
RSD	3.48	2.32	1.83	15.88

*API* active pharmaceutical ingredient, *A* included 105.0 mg of ferrous ions as ferrous sulfate heptahydrate and 350 μg of folic acid in the tablet form with prolonged release of bioactives, *B* contained 100.0 mg of iron as ferrous sulfate heptahydrate and 60 mg of ascorbic acid, similarly as in composition A in the form of tablets with prolonged release, *C* included iron as ferrous gluconate dihydrate corresponding to 23.2 mg of iron, *D* included iron as iron bis-glycinate chelate—28.0 mg, folic acid—400 µg, ascorbic acid—40.0 mg, vitamin B6—1.4 mg and vitamin B12—2.5 mg in the form of capsules, *AAS* atomic absorption spectrometry, *RSD* relative standard deviation

According to Tables 1 and 2, the results of the percentage of released ferrous ions, measured for compositions A, B, and C via conductivity method were more accurate comparing to AAS. Therefore, the conductivity method was further developed, and the validation procedure was initiated for above mentioned compositions. Results obtained in the case of composition D, using both methods, were diversified and not reliable for further analysis. This was confirmed in recommendations which limit the RSD after first hour of analysis to 20%, and at the final time point of dissolution should to 10%. The main hindrance in the development of the conductivity method, and its partial validation was the presence of additional ions, inducing the signal in the solution, e.g., the excipients. Thus, the calibration plots were prepared on the basis of finished products, containing the components potentially available for ionic dissociation in the assessed composition. In addition, the electric conductivity was measured for excipients with high potential signal in the conductivity tests, i.e., folic acid and ascorbic acid. The conductivity measured for folic acid and for ascorbic acid in concentrations exceeding the experiments conditions were 1.06 μS/cm and 39.5 μS/cm, respectively, whereas the controlled conductivity of water was 0.34 μS/cm [20–22].

According to variability of compositions and ferrous ions content, three separate partial validation processes were further evaluated. The ICH guidelines recommend the check of the used system, and maintaining it on the level not higher than 2%. This type of research was considered as pioneer, due to shortage of bibliographic data, thus the suitability of the system (SST) was checked by measuring six times the same sample prepared on 100% of ferrous ions concentration in each composition. The SST data are presented in Table 3.

**Table 3:** System suitability data for the compositions A, B, C assessed via electric conductivity

TN	Electric conductivity/µS cm^−1^		
	Composition A	Composition B	Composition C
1	211.9	340.4	69.43
2	218.4	348.8	68.02
3	220.1	333.3	69.08
4	215.9	340.1	69.60
5	211.6	345.2	68.98
6	210.8	346.8	68.40
Mean	214.8	342.4	68.92
RSD	1.83	1.65	0.88

*TN* test number, *EC* electric conductivity, *RSD* relative standard deviation, codes A, B, and C are due to Table 1

Linearity confirms that the obtained results are directly proportional to the concentration of the active substance at a given level. Linearity was determined by the assessment of ferrous ions percentage on four various levels of concentration, i.e., 60, 80, 100, and 120% for all evaluated drug compositions. The tablet mass was weighed in an appropriate amount, proportional to the required concentrations at levels supplied above (60–120%) for linearity evaluation. Subsequently, the procedure described in section preparation of the test sample was followed. The results in Table 4 are presented as an average of three electric conductivity measurements for each sample on all levels.

**Table 4:** Results of linearity evaluation for the A–C drug formulations containing ferrous ions assessed via electric conductivity

Concentration/%	Formulation		
	A	B	C
	Recovery/%		
60	100.61	97.94	94.64
80	100.43	97.97	90.76
100	100.59	97.49	94.82
120	100.38	97.45	95.84

Codes A, B, and C are due to Table 1

The precision of this method was verified by measuring six individually prepared samples for compositions A, B, and C. The measurement was repeated three times for each sample, and the average result was shown in Table 5. Additionally, intermediate precision was performed on another day using the same method conditions.

**Table 5:** Precision and intermediate precision with RSD data for three evaluated compositions A–C assessed via electric conductivity

Recovery/%						
PM	P	IP	P	IP	P	IP
FT	A		B		C	
SN						
1	98.16	102.64	97.87	95.68	95.02	98.14
2	100.24	101.97	93.57	97.51	93.57	97.64
3	103.54	98.55	97.24	95.37	97.24	97.14
4	100.07	100.45	94.49	95.45	94.49	94.96
5	98.02	102.76	96.37	97.22	96.37	95.14
6	99.64	99.33	94.26	96.62	94.26	94.81
M	99.95	100.95	95.63	96.31	95.16	96.31
RSD	2.00	1.76	1.86	0.97	1.46	1.56
M_12_	100.45		95.97		95.73	
RSD_12_	1.87		1.45		1.57	

Codes A, B, and C are due to Table 1

*PM* precision mode, *P* precision, *IP* intermediate precision, *SN* sample number, *FT* formulation type, *M* mean, *M*_*12*_ mean recovered from P and IP assessment, *RSD* relative standard deviation, *RSD*_*12*_ relative standard deviation recovered from P and IP assessment

There was no possibility to check the accuracy of this method because A, B, and C are the market drugs. Therefore, it would be problematic to prepare a placebo for each of these drug compositions. Hopefully, the future studies will allow to refine the method more accurately. This would lead to preparation of an in-house tablet formulation to perform a full validation process which may confirm the applicability of the method as good alternative to AAS. According to ICH guidelines, evaluation of the limit of quantification (LOQ) and of the limit of detection (LOD) may be omitted, due to high concentrations of ferrous ions in assessed compositions. The robustness was not evaluated in any terms, as the above method is simple, direct, and rapid; there was no observed potent mutability in the performed procedures. The acceptance criteria for dissolution results should be over 80% of released active substance in drug composition, due to the statements of ICH guidelines, and this requirement was fulfilled in our validation procedures.

## Conclusion

The electric conductivity method for evaluation of iron ions release in the conditions congenial to pharmacopoeial standards has been developed, and partially validated. The requirements of ICH guidelines were executed in the case of compositions of ferrous ions, particularly: system suitability, linearity, precision, and intermediate precision. The recorded results were within acceptance criteria, thus the austere conductivity measurements may be further developed as practical alternative to the sophisticated AAS methods.

## Experimental

### Materials

Four various preparations containing ferrous ions were used in the study. Composition A included 105.0 mg of ferrous ions as ferrous sulfate heptahydrate, and 350 µg of folic acid, in the form of tablet with prolonged release of bioactives. Composition B contained 100 mg of iron as ferrous sulfate heptahydrate and 60 mg of ascorbic acid, similarly as in composition A in the form of tablets with prolonged release. Composition C included iron as ferrous gluconate dihydrate corresponding to 23.2 mg of iron. Composition D included iron as iron bis-glycinate chelate—28.0 mg, folic acid—400 µg, ascorbic acid—40.0 mg, vitamin B6—1.4 mg, and vitamin B12—2.5 mg in the form of capsules.

Deionized water from dedicated device (Hydrolab HLP20UV, Straszyn, Poland) was used in all the conductivity measurements, and in the AAS assessments, the AAS calibration working standard solutions were prepared using the iron standard solution of 20 mg/dm^3^ and 0.2% of HNO_3_ (Perkin Elmer, Cracow, Poland), and a solution containing 0.03% Mg as Mg(NO_3_)_2_ and 0.05%Pd as Pd(NO_3_)_2_ was used as a matrix modifier (Perkin Elmer, Cracow, Poland).

### Instrumentation

The dissolution tester Erweka DT 700 (Heusenstamm, Germany) with paddle or basket was used, depending on drug composition, for the evaluation of the formulation dissolution, according to pharmacopoeial standards. Perkin Elmer PinAAcle 900 atomic absorption spectrometer equipped with a THGA graphite furnace (Waltham, MA, USA), Elmetron conductivity meter CC-505 with conductivity probe EC-70 (Elmetron, Zabrze, Poland), and analytical balance AS 160/C/2 (RADWAG, Radom, Poland) were used in analytical procedures.

## Methods

The sample solution preparations for conductivity measurements were obtained from the dissolution studies, performed via dissolution tests using the paddle (compositions A, B, and C), or basket (composition D) at 75 rpm. Evaluated tablets (A, B, C) and capsules (D) were placed on the bottom of dissolution vessels and dissolution test according to the European Pharmacopoeia was performed in this experiment [19]. Purified water of 37 °C as an acceptor medium, in a volume of 900 cm^3^ was used during entire experiment. Conductivity probe was placed in the middle of dissolution vessel, and the results were read and recorded directly from the conductivity meter in µs/cm. The conductivity measurements were performed every 5 min until equilibrium state was reached for each composition: 180 min for compositions A and B, 50 min for composition C, and 75 min for composition D. The analysis was performed in six vessels for each composition. The respective calibration plots for conductivity evaluation were prepared from the entire tablet powders or from capsules filling, to reduce the possibility of errors, resulting from the presence of excipients and additional bioactives.

Samples for AAS were prepared in the same way as samples for conductivity assessment, with exception of retaining 2.0 cm^3^ of each sample, and consequently filtering it through RC 0.45 µm syringe filters (Whatman, Little Chalfont, UK) directly to Eppendorf Safe Lock tubes (Eppendorf, Warsaw, Poland). The acceptor fluid was not supplemented. Time of analysis was the same as in conductivity method. Samples were diluted few times, depending on ions concentration in the composition, and analyzed directly with atomic absorption spectrometer. The calibration working standard solution, prepared from the above mentioned iron standard solution, was in the range of the iron ions concentration 5–25 μg/dm^3^. The calibration plots were prepared using iron standard solutions in 10 and 20 ppb concentrations.

The operating conditions and instrumental parameters for iron determination in samples by GF-AAS are set in Table 6. The mean recovery of iron ions obtained for selected spiked samples was 96.5%.

**Table 6:** The operating conditions and instrumental parameters for iron determination in samples by graphite furnace AAS

Step	Temperature/ °C	Ramp time/s	Hold time/s	Flow rate/cm^3^ min^−1^	Gas type
1	110	1	30	250	Argon
2	130	15	40	250	Argon
3	300	15	5	250	Air
4	550	15	30	250	Air
5	550	1	15	250	Argon
6	1100	5	5	250	Argon
7	1400	10	20	250	Argon
6	2100	0	3.5	0	Argon
7	2450	1	3	250	Argon
Instrumental parameters					
Gas		Argon and air			
Signal type		AA–BG Zeeman correction			
Wavelength		248.33 nm			
Slit width		0.2 nm			
Lamp current		25 mA			
Sample volume		20 mm^3^			
Matrix modifier volume		5 mm^3^ [0.03% Mg as Mg(NO_3_)_2_ and 0.05%Pd as Pd(NO_3_)_2_]			
Measurement mode		Peak area			
Characteristic mass		12.2 pg			

## References

[1] Abbaspour N, Hurrell R, Kelishadi R (2014). J Res Med Sci.

[2] Alleyne M, Horne MK, Miller JL (2008). Am J Med.

[3] Johnson-Wimbley TD, Graham DY (2011). Therap Adv Gastreterol.

[4] Baltussen R, Knai C, Sharan M (2004). J Nutr.

[5] Özdemir N (2015). Turk Pediatri Ars.

[6] Cullen A, O ‘Toole E, Coughlan D, Chalasani K, Leonard T (2013) Pharmaceutical compositions of iron for oral administration. EP 2661273 A2, Nov 13, 2013

[7] Geisser P, Burckhardt S (2011). Pharm.

[8] Santiago P (2012). Sci World J.

[9] Szarfarc SC, de Cassana LM, Fujimori E, Guerra-Shinohara EM, de Oliveira IM (2001). Arch Latinoam Nutr.

[10] Welz B, Sperling M (1999). Atomic absorption spectometry.

[11] Tautkus S, Steponeniene L, Kazlauskas R (2004). J Serb Chem Soc.

[12] Rehber-Turker A, Bag H, Erdogan B (1997). Fresenius J Anal Chem.

[13] Da-ColSemíramis JA, Domene SMA, Pereira-Filho ER (2009). Food Anal Method.

[14] Kojuncu Y, Bundalevska JM, Ay U, Cundeva K, Stafilov T, Akcin G (2004). Sep Sci Technol.

[15] EPA (2012). 5.9 Conductivity. in water: monitoring and assessment. http://water.epa.gov. Accessed 16 Oct 2017

[16] Prieto F, Barrado E, Vega M, Deban L (2001). Russ J Appl Chem.

[17] Regberg A, Singha K, Tien M, Picardal F, Zheng Q, Schieber J, Roden E, Brantley SL (2011). Water Resour Res.

[18] International conference on harmonisation of technical requirements for registration of pharmaceuticals for human use: validation of analytical procedures: text and methodology Q2(R1); Current Step 4 version Parent Guideline dated 27 October 1994 (Complementary Guideline on Methodology dated 6 November 1996 incorporated in November 2005). https://www.ich.org/fileadmin/Public_Web_Site/ICH_Products/Guidelines/Quality/Q2_R1/Step4/Q2_R1__Guideline.pdf. Accessed 16 Oct 2017

[19] Council of Europe. European Pharmacopoeia Commission. Dissolution test for solid dosage forms (Chapter 2.9.3). In: European Pharmacopoeia. 5th edn. Strasburg, Council of Europe, p 20903

[20] Pisoschi AM, Pop A, Serban AI, Fafaneata C (2014). Electrochim Acta.

[21] Wu GH, Wu YF, Liu XW, Rong MC, Chen XM, Chen X (2012). Anal Chim Acta.

[22] Wang C, Li C, Ting L, Xu X, Wang C (2006). Microchim Acta.

